# Immersive display systems for simulating natural vision

**DOI:** 10.64898/2026.07.27.741046

**Published:** 2026-07-30

**Authors:** Harish Katti, Aidan P. Murphy, Michael Helde, Harshawardhan Deshpande, Tyler J. Lee, Rekeya Knight, Carly Gregg, Cristina Solinas, Katherine Cameron, Daryl Bandy, George Dold, David A. Leopold

## Abstract

Vision is traditionally studied using simplified stimuli presented briefly near the center of a flat display while subjects maintain visual fixation. This paradigm contrasts sharply with real-world vision, which is immersive, dynamic and strongly entrained to self-initiated actions. Traditional approaches have allowed researchers to systematically study the neural encoding of visual features and the influence of cognitive operations such as attention. However, other methods are needed to study more holistic and first-person perspectives on vision, such as those related to physical space, continuous time, and self-movement. To enable the study of these and other aspects of real-world vision, we developed hemispherical (“dome”) display systems for macaque visual neuroscience. For functional MRI experiments, a compact rear-projection dome display fits within the bore of a clinical MRI scanner. For electrophysiological recordings, a larger front-projection dome display is illuminated from above using a spherical mirror. Both setups enable complete and dynamic stimulation of approximately 180° of the subject’s field of view, thus facilitating studies requiring visual immersion. To ensure accurate angular geometry across the hemispherical display, we present unified rendering and calibration software that supports natural fisheye videos, conventional visual stimuli, and virtual 3D environments. The calibration procedure automatically compensates for projector, mirror, and dome distortions through geometric pre-warping, ensuring correct visual-angle representation across the display. Pilot fMRI experiments demonstrate robust activation of peripheral visual cortex during both conventional pattern stimulation and naturalistic self-movement. Together, these dome systems provide a flexible platform for investigating aspects of vision that are difficult to study with conventional displays, including peripheral processing, visual immersion, optic flow, self-motion, and holistic scene perception.

## Full Text Availability

The license terms selected by the author(s) for this preprint version do not permit archiving in PMC. The full text is available from the preprint server.

